# Real‐World Dose Adjustment and Switching of Interleukin‐17/23 Inhibitors for Thai Psoriasis

**DOI:** 10.1155/drp/7998583

**Published:** 2026-07-18

**Authors:** Chayada Chaiyabutr, Teerapat Paringkarn, Thanakorn Woramongkol, Thrit Hutachoke, Narumol Silpa-Archa, Leena Chularojanamontri, Chanisada Wongpraparut

**Affiliations:** ^1^ Department of Dermatology, Faculty of Medicine Siriraj Hospital, Mahidol University, Bangkok, Thailand, mahidol.ac.th

**Keywords:** biologics, dose reduction, modification, psoriasis switching

## Abstract

Interleukin (IL)‐17 and IL‐23 inhibitors have transformed psoriasis care, delivering high clearance rates and quality‐of‐life gains. Nevertheless, inadequate response or adverse events can lead to treatment discontinuation or biologic switching. Dose modifications are also commonly implemented in routine practice to optimize efficacy, safety, and cost. The objective of this study is to characterize real‐world outcomes of dose adjustment and switching of IL‐17 and IL‐23 inhibitors in Thai patients with psoriasis. We retrospectively reviewed 173 treatment courses with IL‐17 inhibitors (secukinumab, ixekizumab, and brodalumab) and the IL‐23 inhibitor guselkumab, documenting loading regimens, maintenance dosing, efficacy to Week 52, and switching events. At Week 12, full loading and standard maintenance dosing were associated with higher response rates in most cases. Ixekizumab was least often given with a full loading dose or standard maintenance dosing. By Weeks 24 and 52, reduced‐dose regimens achieved comparable or better outcomes, suggesting careful patient selection. Thirty courses underwent biologic switching, predominantly for secondary failure. Intraclass switching among IL‐17 inhibitors predominated. Switching from IL‐23 to IL‐17 inhibitors potentially outperformed both intraclass IL‐17 switching or IL‐17‐to‐IL‐23 transitions. Erythrodermic psoriasis and higher baseline disease severity predicted switching, whereas incomplete loading doses and dose reductions did not. In conclusion, full loading and standard maintenance regimens facilitate early treatment response, while dose reduction in carefully selected patients can sustain long‐term disease control without increasing the risk of switching.

## 1. Introduction

Psoriasis is a prevalent, acquired, chronic inflammatory dermatosis, with reported prevalence ranging from 0.14% in East Asia to 1.99% in Australasia [1]. No definitive cure exists. Mechanistically, psoriasis is driven by immune dysregulation, mainly involving the interleukin (IL)‐23/IL‐17 axis. IL‐23 promotes the differentiation and maintenance of *T* helper 17 (Th17) cells, which produce IL‐17 and other proinflammatory cytokines that finally stimulate keratinocyte proliferation and sustain chronic inflammation. Targeting this pathway has led to the development of highly effective biologic therapies. IL‐17 inhibitors directly neutralize IL‐17 signaling, whereas IL‐23 inhibitors act upstream by suppressing Th17‐mediated responses, thereby interrupting key pathogenic mechanisms [2].

According to the International Psoriasis Council, patients with psoriasis should be categorized as candidates for either topical or systemic therapy. Candidates for systemic therapy are defined as those meeting at least one of the following criteria: (1) body surface area (BSA) > 10%, (2) involvement of special areas, or (3) inadequate response to topical therapy [3]. In many countries, biologic therapies are indicated following failure of or intolerance to conventional nonbiologic systemic treatments [4, 5]. However, in certain circumstances, such as very severe disease or when an adequate response to conventional agents is not expected, biologics can be used as first‐line systemic therapy, as supported by international guidelines [6]. Furthermore, the American Academy of Dermatology–National Psoriasis Foundation endorse biologics as first‐line options for moderate‐to‐severe plaque psoriasis given their high efficacy and acceptable safety profiles [7].

Biologic agents have revolutionized management by providing targeted treatment that achieves durable disease control, improves quality of life, and maintains favorable safety profiles. IL‐17 and IL‐23 inhibitors are among the most effective biologic therapies currently available [8]. Expert consensus suggests that IL‐17 and IL‐23 inhibitors are preferred over tumor necrosis factor‐α and IL‐12/23 inhibitors as first‐line biologic therapies [5].

Despite their high efficacy, some patients experience suboptimal responses or adverse events that necessitate discontinuation or switching to alternative biologics. In daily practice, clinicians also modify doses—through escalation, reduction, interval extension, or on‐demand regimens—to balance efficacy, cost, safety, and concerns over long‐term exposure [9–11]. Real‐world studies have increasingly captured these dose‐modification strategies together with patterns of biologic switching, reflecting the dynamic adjustments required to optimize treatment outcomes in clinical practice [10, 12, 13]. We therefore aimed to evaluate dose‐modification strategies for IL‐17 and IL‐23 inhibitors and subsequent biologic switching after treatment failure in a Thai real‐world setting.

## 2. Methods

This retrospective study was performed at the Psoriasis Clinic, Department of Dermatology, Faculty of Medicine Siriraj Hospital, Bangkok, Thailand, and received Siriraj Institutional Review Board approval (reference: Si 632/2023). We reviewed records of all psoriasis patients treated with IL‐17 or IL‐23 inhibitors between January 2015 and June 2023. Extracted variables comprised demographics, dosing strategies during loading and maintenance phases, discontinuation and switching patterns, drug survival, and outcomes after switching to other biologics.

Drug survival refers to treatment persistence and was defined as the interval from treatment initiation to discontinuation, with discontinuation defined as a drug‐free gap > 90 days [14]. Dosing was partitioned into loading and maintenance phases. Loading regimens were classified as full (completion of the recommended loading‐phase schedule), partial (incomplete loading‐phase schedule), or absent (no loading‐phase doses administered) [15]. Maintenance regimens were grouped as (1) standard dose (label‐recommended); (2) dose reduction (reduced dose or extended interval); or (3) dose escalation (increased dose or shortened interval) [15]. Standard dosing was defined according to the approved adult prescribing information for each biologic agent [16]. A maintenance change had to persist for ≥ 2 consecutive dosing intervals to qualify as reduction or escalation. The Psoriasis Area and Severity Index (PASI) was used to define response. The primary efficacy outcome was the achievement of PASI 75. Primary failure was the inability to achieve PASI 50 within 3 months. Secondary failure occurred when a patient who had reached PASI 50 at 3 months subsequently relapsed, with PASI rising above 50% of baseline [17]. Switching was defined as discontinuation of the index biologic followed by initiation of another biologic and was determined by the treating physician based on clinical response, adverse events, or nonmedical factors. Intraclass switching was defined as switching between biologics within the same therapeutic class (e.g., IL‐17 to IL‐17), whereas interclass switching referred to switching between different classes (e.g., IL‐17 to IL‐23).

Analyses employed IBM SPSS Statistics Version 29 (IBM Corp, Armonk, NY, USA). A *p* < 0.05 denoted statistical significance. Kaplan–Meier methods estimated drug survival, the log‐rank test compared survival between drugs, and backward elimination regression identified factors associated with switching and postswitch survival.

## 3. Results

### 3.1. Baseline Characteristics

In all, 173 treatment courses with IL‐17 or IL‐23 inhibitors were analyzed: 159 used IL‐17 inhibitors, and 14 used IL‐23 inhibitors (Table 1). Males represented 54.3%, and the mean age was 46.2 years. Chronic plaque‐type psoriasis occurred in 82.1% of cases. Reported comorbidities included psoriatic arthritis (23.8%), dyslipidemia (20.8%), hypertension (17.3%), and diabetes mellitus (16.2%). Approximately 80% of courses involved patients without alcohol or smoking histories. The median baseline PASI score was 12.2. Previous systemic therapy was recorded in 96.4%, most commonly methotrexate (86.1%), cyclosporine (49.4%), and acitretin (47.6%); prior systemic exposure was comparable across biologic groups. Overall, 26.6% of courses followed prior biologic treatment. Ixekizumab had the highest previous biologic exposure (37.2%), whereas brodalumab had the lowest (15.4%).

**TABLE 1:** Baseline demographic and clinical characteristics of 173 psoriasis treatment courses with IL‐17 or IL‐23 inhibitors.

Demographic data	Total drug course (*n* = 173)	Secukinumab (*n* = 103)	Ixekizumab (*n* = 43)	Brodalumab (*n* = 13)	Guselkumab (*n* = 14)
Sex, male, *n* (%)	94 (54.3)	52 (50.5)	26 (60.5)	9 (69.2)	7 (50.0)
Age, years, mean ± SD	46.2 ± 14.4	46.8 ± 13.0	45.9 ± 16.7	42.9 ± 18.0	45.9 ± 14.0
Age at diagnosis, years, mean ± SD	29.6 ± 13.3	28.2 ± 12.2	34.3 ± 15.5	29.9 ± 16.3	27.3 ± 11.1
BMI, kg/m^2^, median + IQR	26.6 + 8.8	26.6 + 7.5	25.2 + 8.1	23.9 + 8.9	31.4 + 8.0
Type of psoriasis, *n* (%)					
‐ Chronic plaque	142 (82.1)	87 (84.5)	34 (79.1)	9 (69.2)	12 (85.7)
‐ Guttate	2 (1.2)	2 (1.9)	—	—	—
‐ Erythroderma	22 (12.7)	13 (12.6)	6 (14.0)	1 (7.7)	2 (14.3)
‐ Generalized pustular psoriasis	5 (2.9)	1 (1.0)	2 (4.7)	2 (15.4)	—
‐ Palmoplantar pustular psoriasis	1 (0.6)	—	—	1 (7.7)	—
‐ Acrodermatitis continua of Hallopeau	1 (0.6)	—	1 (2.3)	—	—
Familial history, *n* (%) (*n* = 91)	30 (33.3)	18 (30.5)	7 (38.9)	2 (40.0)	3 (33.3)
Alcohol consumption, *n* (%) (*n* = 97)					
‐ Never	79 (81.4)	47 (79.7)	17 (89.5)	7 (87.5)	8 (72.7)
‐ Previous	15 (15.5)	10 (16.9)	1 (5.3)	1 (12.5)	3 (27.3)
‐ Current	3 (3.1)	2 (3.4)	1 (5.3)	—	—
Smoking status, *n* (%) (*n* = 98)					
‐ Never	82 (83.7)	49 (81.7)	18 (94.7)	7 (87.5)	8 (72.7)
‐ Previous	10 (10.2)	5 (8.3)	1 (5.3)	1 (12.5)	3 (27.3)
‐ Current	6 (6.1)	6 (10.0)	—	—	—
Comorbidities, *n* (%)					
‐ Diabetes mellitus	28 (16.2)	16 (15.5)	7 (16.3)	2 (15.4)	3 (21.4)
‐ Hypertension	30 (17.3)	24 (23.3)	3 (7.0)	—	3 (21.4)
‐ Dyslipidemia	36 (20.8)	27 (26.2)	6 (14.0)	1 (7.7)	2 (14.3)
‐ Psoriatic arthritis (*n* = 172)	41 (23.8)	29 (28.2)	8 (19.0)	1 (7.7)	3 (21.4)
‐ Cardiovascular disease	4 (2.3)	2 (1.9)	2 (4.7)	—	—
‐ Tuberculosis	5 (2.9)	3 (2.9)	1 (2.3)	1 (7.7)	—
‐ Inflammatory bowel disease	—	—	—	—	—
‐ Psychiatric disease	9 (5.2)	5 (4.9)	1 (2.3)	1 (7.7)	2 (14.3)
‐ Malignancy	6 (3.5)	3 (2.9)	3 (7.0)	—	—
Special areas, *n* (%) (*n* = 166)	161 (97.0)	98 (97.0)	38 (97.4)	13 (100.0)	12 (92.3)
‐ Scalp	138 (83.1)	84 (83.2)	30 (76.9)	12 (92.3)	12 (92.3)
‐ Nail	104 (62.7)	65 (64.4)	25 (64.1)	5 (38.5)	9 (69.2)
‐ Palmoplantar	34 (20.5)	24 (23.8)	4 (10.3)	2 (15.4)	4 (30.8)
‐ Inverse	35 (21.1)	24 (23.8)	5 (12.8)	1 (7.7)	5 (38.5)
‐ Genital	17 (10.2)	10 (9.9)	3 (7.7)	1 (7.7)	3 (23.1)
Systemic experienced, *n* (%) (*n* = 168)	162 (96.4)	98 (97.0)	38 (95.0)	13 (100.0)	14 (100.0)
‐ Methotrexate (*n* = 166)	143 (86.1)	89 (89.0)	32 (80.0)	11 (91.7)	11 (78.6)
‐ Cyclosporine (*n* = 166)	82 (49.4)	58 (58.0)	12 (30.0)	4 (33.3)	8 (57.1)
‐ Acitretin (*n* = 166)	79 (47.6)	52 (52.0)	15 (37.5)	7 (58.3)	5 (35.7)
Biologic experienced, *n* (%)	46 (26.6)	23 (22.3)	16 (37.2)	2 (15.4)	5 (35.7)
‐ TNF‐α inhibitors	15 (8.7)	13 (12.6)	1 (2.3)	—	1 (7.1)
‐ IL‐12/23 inhibitors	1 (0.6)	1 (1.0)	—	—	—
‐ IL‐17 inhibitors	25 (14.5)	7 (6.8)	12 (27.9)	2 (15.4)	4 (28.6)
‐ IL‐23 inhibitors	5 (2.9)	2 (1.9)	3 (7.0)	—	—
Phototherapy experienced, *n* (%) (*n* = 158)					
‐ Narrowband ultraviolet B	80 (50.6)	53 (53.5)	18 (48.6)	6 (50.0)	3 (30.0)
‐ Psoralen–ultraviolet A	31 (19.6)	22 (22.2)	5 (13.5)	1 (8.3)	3 (30.0)
Baseline PASI, median + IQR (*n* = 122)	12.2 + 10.4	12.2 + 10.1	11.0 + 13.3	9.9 + 9.6	17.5 + 11.7
Loading dose, *n* (%) (*n* = 167)					
‐ No loading	53 (31.7)	35 (34.7)	15 (38.5)	2 (15.4)	1 (7.1)
‐ Partial loading	45 (26.9)	22 (21.8)	19 (48.7)	4 (30.8)	—
‐ Full loading	69 (41.3)	44 (43.6)	5 (12.8)	7 (53.8)	13 (92.9)
Maintenance phase, *n* (%) (*n* = 135)					
‐ Standard dose	57 (42.2)	36 (41.4)	9 (33.3)	6 (50.0)	6 (66.7)
‐ Dose escalation	5 (3.7)	5 (5.7)	—	—	—
‐ Dose reduction	73 (54.1)	46 (52.9)	18 (66.7)	6 (50.0)	3 (33.3)

*Note:* Values are presented as *n* (%) unless otherwise indicated. IL, interleukin; IQR, interquartile range.

Abbreviations: BMI, body mass index; PASI, Psoriasis Area and Severity Index; SD, standard deviation; TNF, tumor necrosis factor.

### 3.2. Treatment Effectiveness

**FIGURE 1:**
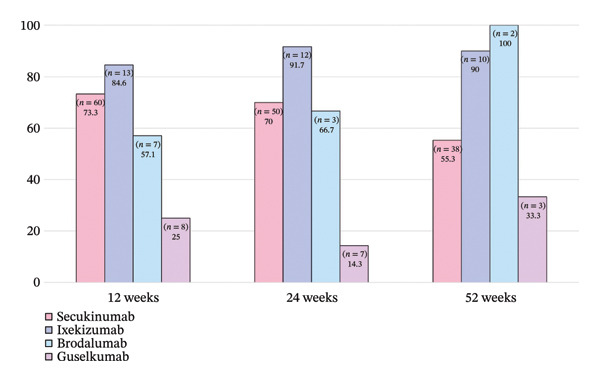
Proportion of patients achieving PASI 75 at Weeks 12, 24, and 52, stratified by biologic agent.

**FIGURE 2:**
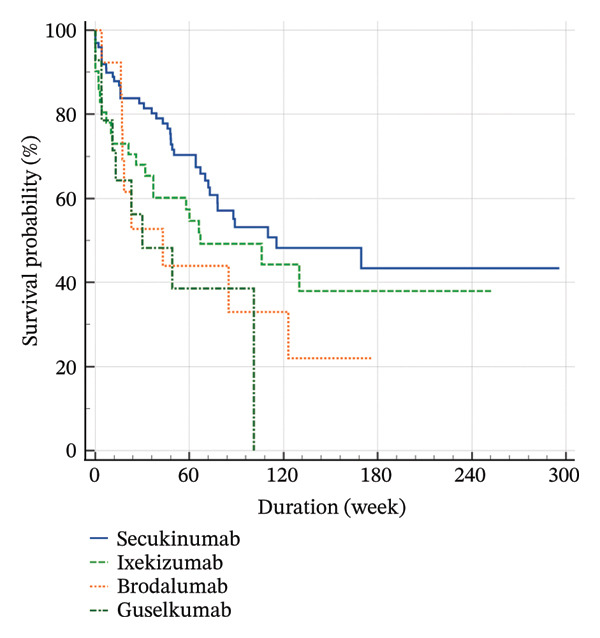
Kaplan–Meier drug‐survival curves for individual IL‐17 and IL‐23 inhibitors.

Effectiveness was assessed by PASI 75 achievement (Figure 1). Ixekizumab produced the highest response at Week 12 (84.6%) and Week 24 (91.7%), followed by secukinumab (73.3% and 70%, respectively). At Week 52, brodalumab yielded 100% PASI 75, with ixekizumab next at 90%. When assessed using PASI 90 (Figure S1), ixekizumab showed the highest response at Week 12 (61.5%), whereas brodalumab demonstrated the highest responses at Weeks 24 and 52. Drug survival differed significantly between biologics (Figure 2; *p* = 0.0374); secukinumab showed the longest survival, followed by ixekizumab, brodalumab, and guselkumab.

### 3.3. Loading‐Dose Analysis

**FIGURE 3:**
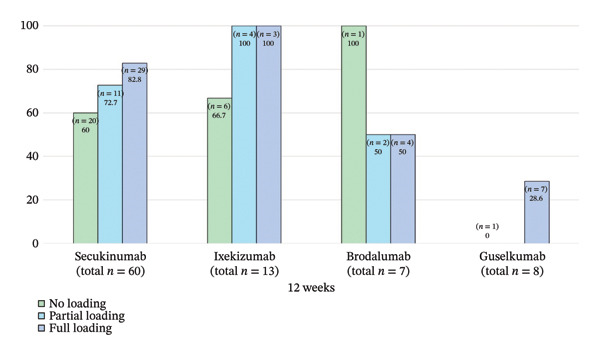
Week‐12 PASI 75 response according to the loading‐dose strategy.

Overall, 41.3% of patients received the full loading dose, 26.9% received a partial loading dose, and 31.7% received none. Full loading was most frequent with guselkumab (92.9%) and brodalumab (53.8%), whereas ixekizumab accounted for most partial or absent loading regimens (Table 1). Within secukinumab, PASI 75 at Week 12 was 82.8% with full loading, 72.7% with partial loading, and 60% with no loading (Figure 3). Ixekizumab achieved PASI 75 in 100% of patients with either full or partial loading. Conversely, only 28.6% of guselkumab courses with full loading reached PASI 75, and brodalumab exhibited its highest response in patients without loading; interpretation is limited by small sample sizes of guselkumab and brodalumab. When assessed by PASI 90 (Figure S2), ixekizumab maintained high response rates, with PASI 90 achieved predominantly in patients receiving full loading.

### 3.4. Maintenance‐Dose Analysis

**FIGURE 4:**
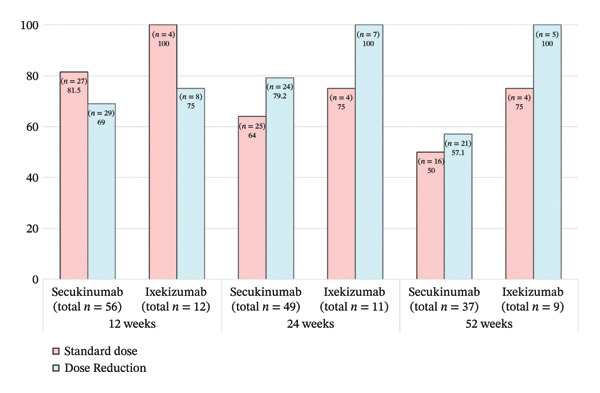
PASI 75 response at Weeks 12, 24, and 52 according to the maintenance‐dose strategy.

During maintenance, 42.2% received label‐recommended dosing, 54.1% underwent dose reduction, and 3.7% underwent dose escalation (Table 1). Ixekizumab had the lowest on‐label use (33.3%) and the highest reduction rate (66.7%), whereas guselkumab had the highest on‐label maintenance use (66.7%). When categorizing effectiveness based on the maintenance phase (Figure 4), we analyzed only the standard‐dose and dose‐reduction groups for secukinumab and ixekizumab, as the other drugs and the dose‐escalation group had very small patient numbers. At Week 12, standard maintenance dosing with secukinumab and ixekizumab resulted in higher PASI 75 responses compared to dose reduction. However, by Weeks 24 and 52, the outcomes in the dose‐reduction groups matched or surpassed standard dosing at Weeks 24 and 52. When assessed by PASI 90 (Figure S3), dose reduction demonstrated comparable or higher response rates than the standard regimen.

### 3.5. Switching Patterns

Thirty switches occurred among the 173 courses (Table 2). Secondary failure accounted for 23.3% of switches and primary failure for 13.3%, whereas personal or economic considerations explained the remainder; no switch was prompted by adverse events. Intraclass switching within IL‐17 inhibitors predominated (70%), led by secukinumab‐to‐ixekizumab (30%) and the reciprocal switch (20%); intraclass switches were usually the first switching event in our cohort.

**TABLE 2:** Patterns and frequency of biologic switching among 173 psoriasis treatment courses (*N* = 30 switches).

Class switching		*N* (%)	First switching	Second switching	Third switching
Total		30 (100.0)	24 (80.0)	5 (16.7)	1 (3.3)
IL‐17 to IL‐17 inhibitors		21 (70.0)	20 (95.2)	1 (4.8)	—
IL‐17 to IL‐23 inhibitors		4 (13.3)	1 (25.0)	3 (75.0)	—
IL‐23 to IL‐17 inhibitors		5 (16.7)	3 (60.0)	1 (20.0)	1 (20.0)

**First biologic**	**Second biologic**				

Secukinumab	Ixekizumab	9 (30.0)	9 (100.0)	—	—
Secukinumab	Guselkumab	2 (6.7)	1 (50.0)	1 (50.0)	—
Ixekizumab	Secukinumab	6 (20.0)	6 (100.0)	—	—
Ixekizumab	Brodalumab	2 (6.7)	1 (50.0)	1 (50.0)	—
Ixekizumab	Guselkumab	2 (6.7)	—	2 (100.0)	—
Brodalumab	Secukinumab	2 (6.7)	2 (100.0)	—	—
Brodalumab	Ixekizumab	2 (6.7)	2 (100.0)	—	—
Guselkumab	Secukinumab	2 (6.7)	1 (50.0)	1 (50.0)	—
Guselkumab	Ixekizumab	3 (10.0)	2 (66.7)	—	1 (33.3)

*Note:* IL‐17, interleukin‐17 inhibitor; IL‐23, interleukin‐23 inhibitor; “First biologic” and “Second biologic” denote the initial and subsequent agents, respectively.

### 3.6. Postswitch Drug Survival

**FIGURE 5:**
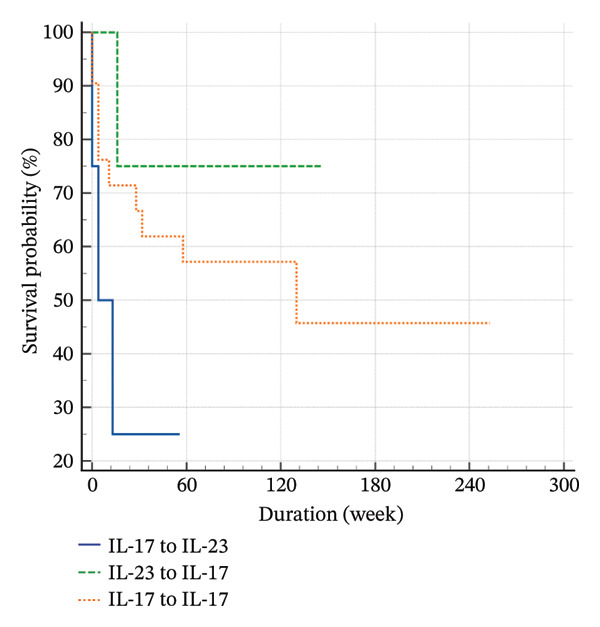
Postswitch drug survival by biologic class.

**FIGURE 6:**
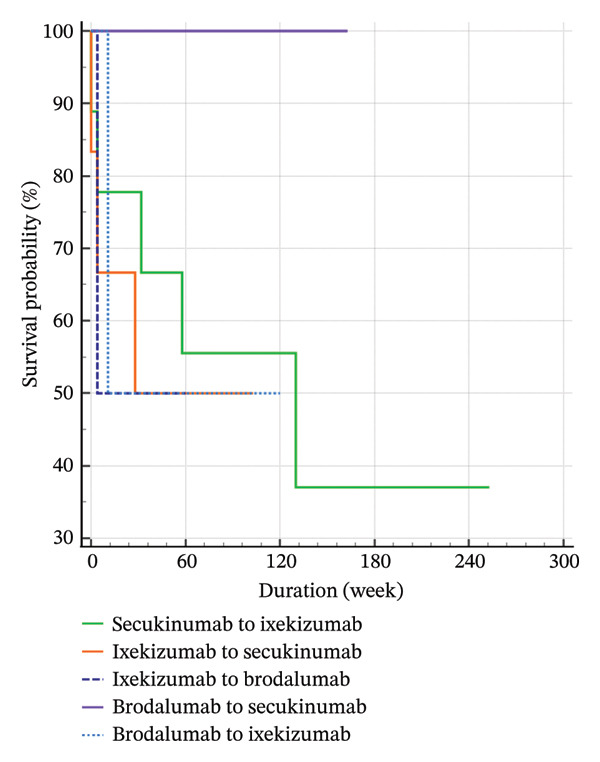
Postswitch drug survival after intraclass switching among IL‐17 inhibitors.

Postswitch survival analysis (Figure 5) showed the best outcomes when switching from IL‐23 to IL‐17 inhibitors, intermediate survival with IL‐17 intraclass switches, and the poorest survival when switching from IL‐17 to IL‐23. Differences were not statistically significant (log‐rank *p* = 0.14). Within the IL‐17 class, switching from brodalumab to secukinumab provided the longest postswitch survival, though differences among intraclass switches were also not statistically significant (Figure 6; *p* = 0.73).

### 3.7. Predictors of Switching and Survival

Univariate analyses identified elevated baseline PASI, greater BSA, and erythrodermic psoriasis as predictors of switching, whereas inverse and pustular variants were linked to shorter postswitch survival (Table S1). Multivariate models confirmed erythrodermic psoriasis as a significant predictor of switching, while scalp involvement and switches from IL‐23 to IL‐17 inhibitors conferred superior postswitch survival. Neither incomplete loading nor maintenance dose reduction influenced switching decisions.

## 4. Discussion

Thailand is an upper‐middle‐income country where biologic therapy is reimbursed only for severe psoriasis, defined as PASI or BSA ≥ 15, in patients who are unresponsive to at least two conventional treatment modalities from the following: methotrexate, cyclosporine, acitretin, and phototherapy, and solely through the Civil Servant Medical Benefit Scheme (a government health insurance program) [4]. This scheme covers < 10% of the population [18]. Among IL‐17/IL‐23 inhibitors, secukinumab is the lone drug on the scheme’s formulary at the time of data collection; ixekizumab, brodalumab, and guselkumab must be funded out of pocket or by private insurance. These policies explain the predominance of secukinumab in Thai practice.

Despite these reimbursement limitations, the demand for biologic therapies is increasing as awareness of their efficacy and safety grows among both clinicians and patients. As many patients must rely on out‐of‐pocket payments. Dose optimization strategies, such as dose reduction or interval extension, are commonly employed in clinical practice to balance treatment effectiveness and cost.

We examined 173 treatment courses and documented 3 principal patterns. First, only 41.3% of patients received a full loading regimen, and maintenance‐phase dose reduction occurred in 54.1%. Second, dose escalation was rare (5.7%) and limited to secukinumab. Third, 30 switches (17.3%) were recorded, driven mainly by secondary failure (23.3%). Intraclass switching among IL‐17 inhibitors accounted for 70% of all changes. The most common transitions were from secukinumab to ixekizumab (30%) and from ixekizumab to secukinumab (20%); intraclass switches were usually the first switching event in our cohort. No switch was attributed to adverse events.

Most tapering studies begin when patients sustain a PASI ≤ 5, a Dermatology Life Quality Index ≤ 5, or a Physician Global Assessment ≤ 1 for ≥ 6 month. Dose is then reduced stepwise, usually by extending intervals to 67% and subsequently 50% of label frequency, or by on‐demand regimens. A lack of clear guidelines and long‐term data remains the main barrier [19]. A 347‐patient Spanish cohort reported that 18.2% underwent secukinumab tapering within 2 years; 77.8% succeeded, survival was unchanged, and drug expenditure fell by 33% [20]. Moreover, in an Italian cohort of 1144 patients treated with IL‐23 or IL‐17 inhibitors, 92.9% maintained PASI ≤ 1 at 12 months after dose spacing. Mean PASI remained stable over time without significant changes, and 70% of patients persisted with the dose‐spacing strategy at 1 year [11]. In a Thai cohort of 142 patients given IL‐17 inhibitors, off‐label schedules matched on‐label regimens for clearance but produced more flares [21].

Our data mirrored the patterns reported in the literature. At Week 12, full or partial loading of secukinumab and ixekizumab and on‐label maintenance, yielded higher PASI 75 rates than no loading or reduced dosing. By Weeks 24 and 52, the trend inverted: Patients in the dose‐reduction group tended to outperform the on‐label group, probably because early high responders were preferentially tapered, creating selection bias. Neither incomplete loading nor maintenance‐phase reduction was associated with later biologic switching.

Several studies report fewer or no adverse events during tapering, and reduced dosing of agents such as adalimumab and ustekinumab has not increased immunogenicity, as judged by the absence of elevated antidrug antibodies [19]. Our findings, including the lack of safety‐related switches, reinforce dose reduction as a viable and safe cost‐containment measure in carefully selected patients.

Dose escalation was reported to be more common among IL‐17 inhibitors than IL‐23 inhibitors. Analyses conducted in the United States show escalation during the full maintenance period in 2.0% of risankizumab and 7.2% of guselkumab courses, versus 15.7% and 18.0% for secukinumab and ixekizumab, respectively [22]. Japanese data report 37.2% escalation for ixekizumab and 3.4% for secukinumab within 12 months [23]. Escalation was generally unlinked to safety concerns [24]. Our 5.7% escalation rate, restricted to secukinumab, aligns with these international observations and underscores the relative stability of IL‐23 inhibitor dosing.

Higher baseline PASI, greater BSA, and erythrodermic psoriasis predicted switching in our univariate analysis; erythrodermic disease remained independent in multivariate models. These findings are consistent with previous studies [25–27]. In the North American CorEvitas Psoriasis Registry, efficacy loss explained 51.7% of 159 switches, and switchers entered with more severe disease [28]. Tokyo data showed multiple switches in 27.3% of 285 patients, with higher PASI and greater psoriatic arthritis prevalence than nonswitchers [29].

In psoriasis switching management, the pattern of response—primary or secondary nonresponse—guides treatment decisions. Primary nonresponse often warrants switching to a different biologic class, while secondary nonresponse may be managed with intraclass switching before considering class change. Many previous studies suggesting that both intra‐ and interclass switching strategies between IL‐17 and IL‐23 inhibitors can be effective for patients with psoriasis in clinical practice.

Evidence supports the effectiveness of within‐class switching among both IL‐17 and IL‐23 inhibitors, due to differences in molecular structure, cytokine selectivity, and binding affinity. Agents such as bimekizumab and brodalumab, which target additional IL‐17 isoforms beyond IL‐17A, may offer improved outcomes in patients unresponsive to earlier IL‐17A inhibitors like secukinumab. Similarly, the higher IL‐17A binding affinity of ixekizumab may underlie its superior efficacy in certain intraclass transitions. Intraclass switching among IL‐17 inhibitors demonstrates favorable clinical outcomes in many studies. A recent meta‐analysis indicated that the proportions of patients achieving reductions in the PASI of 75%, 90%, and 100% were estimated at 74.6%, 69.4%, and 46.4%, respectively, after short‐term treatment of intraclass switching of IL‐17 inhibitors [30, 31].

The limitation of this study is the limited accessibility of biologics in Thailand, yielding a modest sample size. Reimbursement rules confined drug choice, and higher‐priced agents were reserved as second‐line therapies, introducing selection bias. Nonetheless, the findings reflect real‐world practice in resource‐limited settings. Missing follow‐up PASI values curtailed outcome analyses for some regimens.

In conclusion, real‐world data from a resource‐limited setting show that full loading accelerates early clearance, but maintenance‐phase dose reduction sustains control and limits expense. Neither tapering nor incomplete loading precipitated switches. Switching from IL‐23 to IL‐17 inhibitors potentially outperformed both intraclass IL‐17 switching or IL‐17‐to‐IL‐23 transitions. These findings can inform clinicians, payers, and policymakers seeking to optimize psoriasis care under financial constraints.

## Author Contributions

Chayada Chaiyabutr: conceptualization/design; Chayada Chaiyabutr, Leena Chularojanamontri, and Chanisada Wongpraparut: methodology of the study; Teerapat Paringkarn and Thrit Hutachoke: data collection; Thanakorn Woramongkol and Chayada Chaiyabutr: data analysis; Chayada Chaiyabutr: writing–original draft of the manuscript; Chanisada Wongpraparut: writing and editing the manuscript. Narumol Silpa‐Archa and Leena Chularojanamontri: editing and reviewing the manuscript.

## Funding

The authors received no financial support for the research, authorship, and/or publication of this article.

## Disclosure

This research was previously presented as a poster at the 2025 American Academy of Dermatology Annual Meeting (March 7–11, 2025; Orlando, FL, USA).

## Conflicts of Interest

Chayada Chaiyabutr reports honoraria as a speaker from Zuellig Pharma, Novartis, Janssen, and Kyowa Kirin and investigator–consultant from Novartis.

Narumol Silpa‐Archa reports honoraria as a speaker from Zuellig Pharma, Novartis, Janssen, and Kyowa Kirin, and investigator–consultant from Novartis.

Leena Chularojanamontri reports honoraria as a speaker from Zuellig Pharma, Novartis, Janssen, Pfizer, Ranbaxy, and Kyowa Kirin; consultancy/advisory‐board from Zuellig Pharma, Novartis, Janssen, and Ranbaxy; and investigator–consultant from Novartis.

Chanisada Wongpraparut reports honoraria as a speaker from Zuellig Pharma, Novartis, Janssen, Ranbaxy, and Kyowa Kirin, and investigator–consultant from Novartis.

The other authors declare no conflicts of interest.

## Supporting Information

Additional supporting information can be found online in the Supporting Information section.

## Supporting information


**Supporting Information 1** Table S1. Factors associated with drug switching and survival of postswitching biologics.

**Supporting Information 2** Figure S1. Proportion of patients achieving PASI 90 at Weeks 12, 24, and 52, stratified by biologic agent. Figure S2. Week‐12 PASI 90 response according to the loading‐dose strategy. Figure S3. PASI 90 response at Weeks 12, 24, and 52 according to the maintenance‐dose strategy.

## Data Availability

The data that support the findings of this study are available from the corresponding author upon reasonable request.
