# Supplementary material for: Real‐World Dose Adjustment and Switching of Interleukin‐17/23 Inhibitors for Thai Psoriasis
Source: Dermatol Res Pract. 2026 Jul 18;2026:7998583. doi: 10.1155/drp/7998583 (PMC13379887; doi:10.1155/drp/7998583)
Supplement: Supplementary file 1 — Supporting Information 1 Table S1. Factors associated with drug switching and survival of postswitching biologics. [file DRP-2026-7998583-s001.docx]

# Table S1. Factors associated with drug switching and survival of post-switching biologics

|  | drug switching | | | | survival of post-switching biologics | | | |
| --- | --- | --- | --- | --- | --- | --- | --- | --- |
| Factors | Univariable analysis | | Multivariable analysis | | Univariable analysis | | Multivariable analysis | |
|  | Crude OR | *P*-value | Adjusted OR | *P*-value | Crude HR | *P*-value | Adjusted HR | *P*-value |
| Sex: Female | 0.671 (0.285-1.580) | 0.361 |  |  | 1.074 (0.372-3.100) | 0.895 |  |  |
| Age at diagnosis | 1.023 (0.986-1.061) | 0.220 |  |  | 0.951 (0.900-1.005) | 0.077 | 0.968 (0.935-1.002) | 0.063 |
| Age start drug | 1.007 (0.976-1.038) | 0.678 |  |  | 0.980 (0.950-1.012) | 0.216 |  |  |
| Body mass index | 1.051 (0.945-1.168) | 0.361 |  |  | 0.975 (0.811-1.171) | 0.784 |  |  |
| Family history | 1.125 (0.309-4.099) | 0.858 |  |  | 2.760 (0.613-12.427) | 0.186 |  |  |
| Alcohol drinking | 0.738 (0.148-3.675) | 0.711 |  |  | 0.641 (0.080-5.160) | 0.676 |  |  |
| Smoking | 0.955 (0.187-4.863) | 0.955 |  |  | 0.641 (0.080-5.160) | 0.676 |  |  |
| Psoriatic arthritis | 1.272 (0.509-3.176) | 0.607 |  |  | 0.659 (0.184-2.364) | 0.522 |  |  |
| Hypertension | 0.440 (0.124-1.562) | 0.204 |  |  | 0.738 (0.096-5.682) | 0.771 |  |  |
| Diabetes mellitus | 2.346 (0.870-6.330) | 0.092 | 3.649 (0.972-13.695) | 0.055 | 0.655 (0.183-2.352) | 0.517 |  |  |
| Dyslipidemia | 0.525 (0.170-1.627) | 0.264 |  |  | 0.746 (0.167-3.338) | 0.701 |  |  |
| Cardiovascular | 1.360 (0.146-12.679) | 0.787 |  |  | - | - |  |  |
| Psychiatric | 2.833 (0.491-16.337) | 0.244 |  |  | 2.505 (0.544-11.530) | 0.238 |  |  |
| Cancer | 1.360 (0.146-12.679) | 0.787 |  |  | - | - |  |  |
| Scalp | 3.490 (0.779-15.636) | 0.102 |  |  | 0.350 (0.076-1.601) | 0.176 | 0.157 (0.027-0.907) | 0.039* |
| Nail | 2.338 (0.874-6.251) | 0.091 |  |  | 1.222 (0.335-4.462) | 0.761 |  |  |
| Palmoplantar | 1.758 (0.661-4.676) | 0.258 |  |  | 0.822 (0.225-3.007) | 0.767 |  |  |
| Inverse | 0.656 (0.209-2.060) | 0.471 |  |  | 4.343 (1.209-15.603) | 0.024* |  |  |
| Genital | 1.363 (0.358-5.193) | 0.650 |  |  | 3.404 (0.872-13.284) | 0.078 |  |  |
| Baseline PASI | 1.063 (1.014-1.115) | 0.011* |  |  | 0.998 (0.926-1.077) | 0.968 |  |  |
| Baseline BSA | 1.022 (1.006-1.038) | 0.008* |  |  | 0.985 (0.956-1.016) | 0.336 |  |  |
| Loading  - No loading  - Partial loading  - Full loading | Reference  6.458 (0.787-52.986)  7.196 (0.898-57.646) | 0.082  0.063 |  |  | Reference  1.098 (0.234-5.140)  1.602 (0.430-5.966) | 0.906  0.482 |  |  |
| Maintenance  - Standard dose  - Dose reduction | Reference  0.793 (0.267 – 2.349) | 0.675 |  |  | Reference  1.259 (0.208-7.637) | 0.801 |  |  |
| Type   - Chronic plaque type - Erythrodermic - Pustular | Reference  5.315 (1.761-16.041)  3.417 (0.295-39.629) | 0.003*  0.326 | 8.743 (2.075-36.833)  11.738 (0.648-212.488) | 0.003*  0.096 | Reference  1.515 (0.454-5.050) 8.865 (1.606-48.930) | 0.499  0.012* |  |  |
| Class switching   - IL-17i to IL-17i - IL-17i to IL-23i - IL-23i to IL-17i |  |  |  |  | Reference  2.744 (0.720-10.455)  0.443 (0.056-3.480) | 0.139  0.439 | Reference  0.121 (0.011-1.316)  0.215 (0.049-0.944) | 0.083  0.042* |

Abbreviation: BSA-body surface area; HR-hazard ratio; IL-interleukin; OR-Odds ratio; PASI-psoriasis area severity index
