# Supplementary material for: Real‐World Dose Adjustment and Switching of Interleukin‐17/23 Inhibitors for Thai Psoriasis
Source: Dermatol Res Pract. 2026 Jul 18;2026:7998583. doi: 10.1155/drp/7998583 (PMC13379887; doi:10.1155/drp/7998583)

Figure S1. Proportion of Patients Achieving PASI 90 at Weeks 12, 24, and 52, Stratified by Biologic Agent


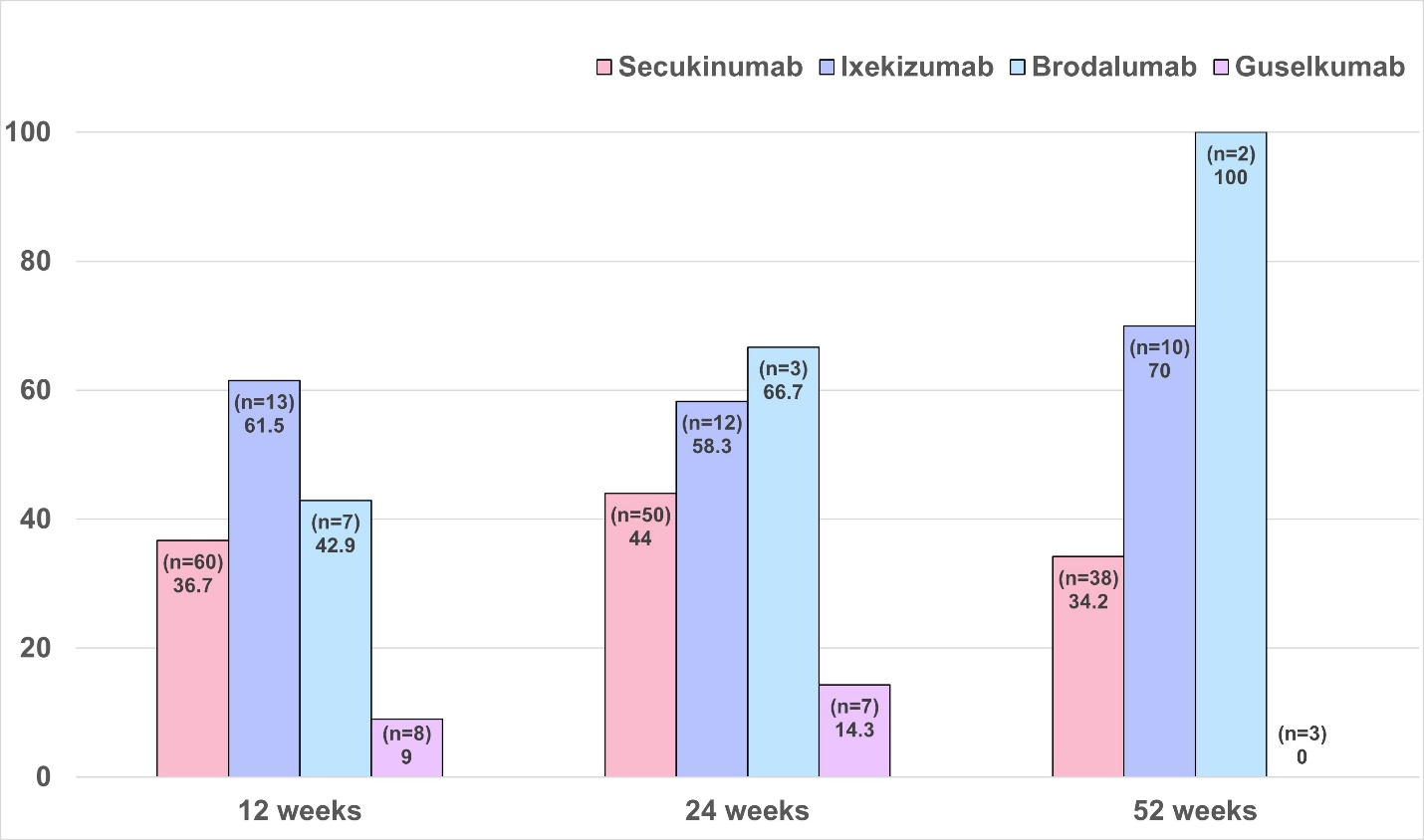


**Figure S2.** Week-12 PASI 90 Response According to Loading-Dose Strategy


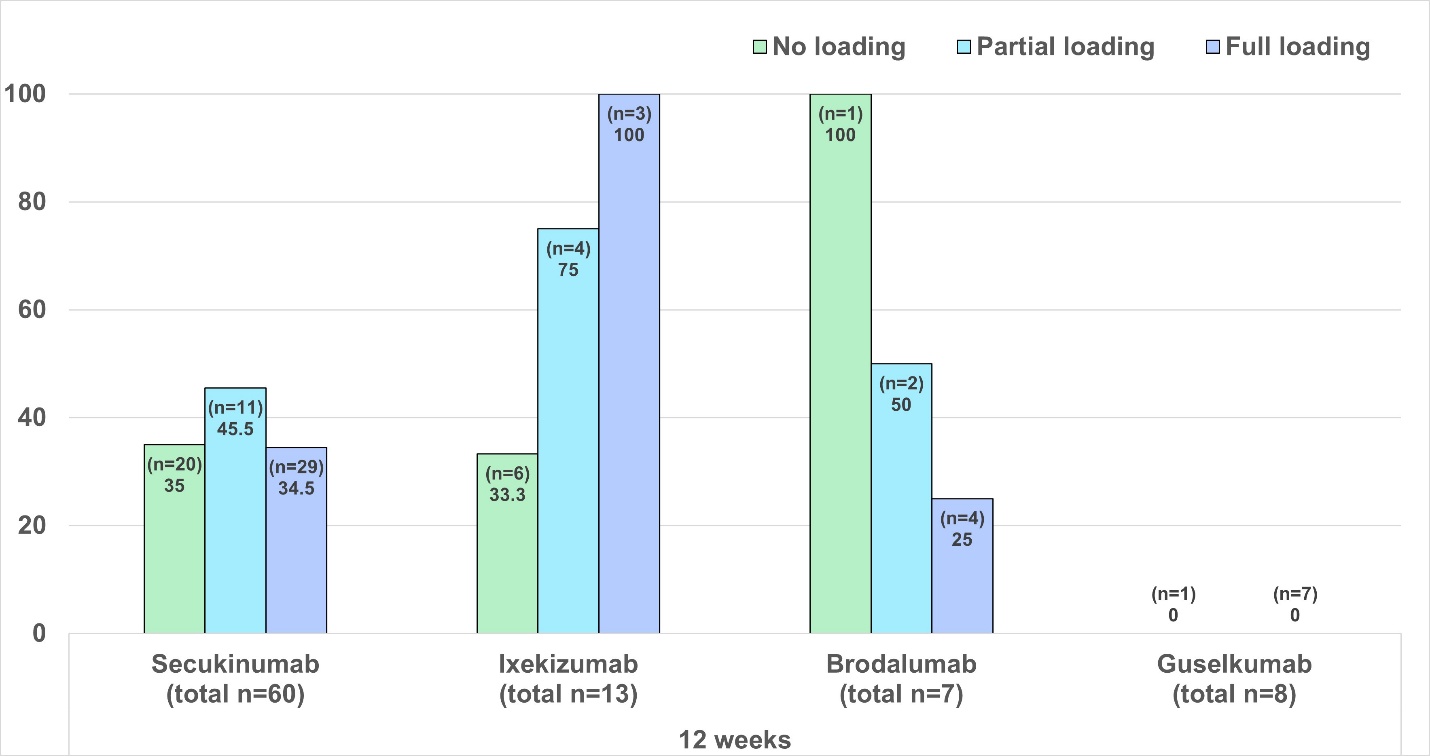


**Figure S3.** PASI 90 Response at Weeks 12, 24, and 52 According to Maintenance-Dose Strategy


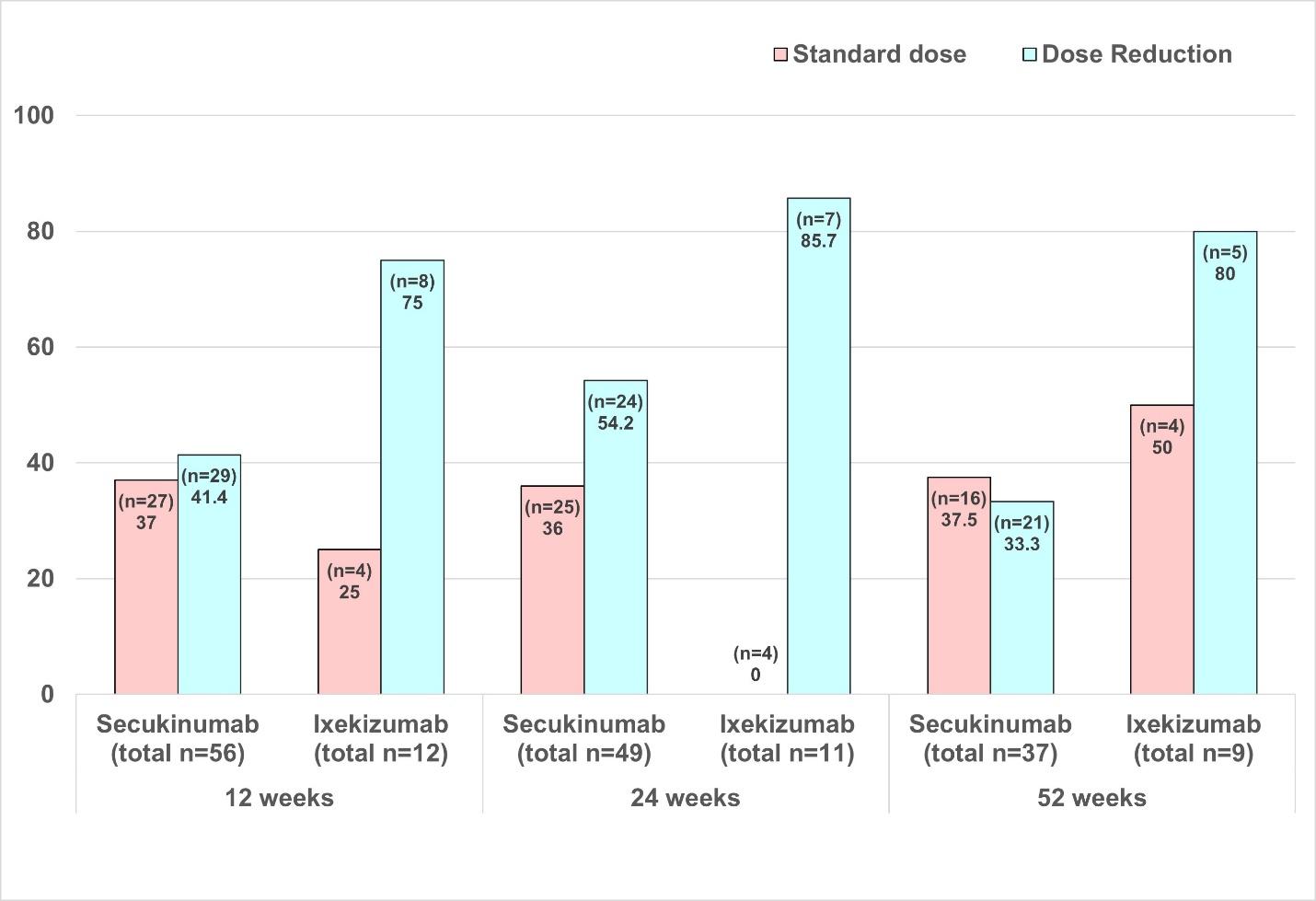

Supplement: Supplementary file 2 — Supporting Information 2 Figure S1. Proportion of patients achieving PASI 90 at Weeks 12, 24, and 52, stratified by biologic agent. Figure S2. Week‐12 PASI 90 response according to the loading‐dose strategy. Figure S3. PASI 90 response at Weeks 12, 24, and 52 according to the maintenance‐dose strategy. [file DRP-2026-7998583-s002.docx]
